# Evolutionary Trajectories of Consciousness: From Biological Foundations to Technological Horizons

**DOI:** 10.3390/brainsci15070734

**Published:** 2025-07-09

**Authors:** Evgenii Gusev, Alexey Sarapultsev, Maria Komelkova

**Affiliations:** 1 Institute of Immunology and Physiology, Ural Branch of the Russian Academy of Science, 106 Pervomaiskaya Street, Ekaterinburg 620049, Russia; a.sarapultsev@gmail.com; 2 Russian–Chinese Education and Research Center of System Pathology, South Ural State University, 76 Lenin Prospekt, Chelyabinsk 454080, Russia; mkomelkova@mail.ru

**Keywords:** consciousness evolution, subjectivity, biological consciousness, social consciousness, neurobiological systems, human cognition, information theory, evolutionary psychology, artificial intelligence, technological subjectivity, ethical considerations

## Abstract

Consciousness remains one of the most critical yet least understood functions of the brain, not only in humans but also in certain highly organized animal species. In this review, we propose treating consciousness as an emergent, goal-directed informational system organized by the subjective “self” as an active system-forming factor. We present an integrative theoretical–systems framework in which subjectivity functions as system-forming factor of consciousness (SFF) throughout biological evolution. Beginning with proto-conscious invertebrates, we trace progressive elaborations of working and long-term memory, the refinement of behavioral programs, and the emergence of an internal arbiter capable of resolving competing drives. In endothermic vertebrates, subjectivity acquires distinct functional features—sensory filtering, causal reasoning, and adaptive arbitration—underpinned by increasingly complex neural architectures. This evolutionary trajectory culminates in humans, where subjectivity attains its highest level of organization through culturally mediated networks. Although the framework does not assume any specific neural substrate, it provides a testable roadmap linking evolutionary biology, information theory, and quantitative modeling. By clarifying why consciousness arose and how subjectivity shapes complex networks, this perspective also lays the groundwork for exploring possible nonbiological extensions of subjectivity.

## 1. Introduction

Consciousness is one of the most immediate yet deeply enigmatic phenomena encountered by humans. The scientific literature on its nature spans a wide array of disciplines—medicine, philosophy, physics, ethology, psychology, neurobiology, neurophysiology, mathematics, and computer science—each contributing distinct methodological approaches to its study. Over decades, researchers have proposed dozens of prominent theories and conceptual frameworks to model consciousness. Examples include Adaptive Resonance Theory (ART), Attention Schema Theory (AST), Dynamic Core Theory (DCT), Global Workspace Theory (GWT) and its neural extension, the Global Neuronal Workspace (GNW), Integrated Information Theory (IIT), Recurrent Processing Theory (RPT), Predictive Processing (PP), Dendritic Integration Theory (DIT), and various electromagnetic, quantum, or psychodynamic accounts. Other notable proposals encompass Apical Dendrite Theory (ADT), Electromagnetic Field Hypotheses (EM/CEMI), Memory, Consciousness, and Temporality Theory (MCTT), Radical Plasticity Thesis (RPT as a distinct formulation), Semantic Pointer Competition (SPC), and numerous network- or circuit-based hypotheses [1,2,3,4,5,6,7,8,9,10,11,12,13,14,15,16,17,18,19,20,21,22,23,24,25,26,27,28,29,30,31,32,33,34,35]. Recent evolutionary-grounded perspectives—neurobiological naturalism (Feinberg & Mallatt, 2016–2022)—and complementary frameworks such as the Dynamic Organicity Theory (Poznanski, 2024) have further enriched this landscape [13,14,15,16,17,25].

Additionally, several review articles, such as those by Sattin [36], Storm [37], and Seth [38], have systematically categorized these existing theories, addressing key issues in consciousness research. Storm J.F. and colleagues integrated six closely related theories of consciousness (GNWT, IIT, RPT, PP, NREP, DIT) into a single conceptual framework. This comparative approach highlights the fact that different theories generally focus on specific aspects or subtypes of “consciousness”, which can lead to conceptual confusion and misinterpretations [37]. Nonetheless, these theories consistently define conscious experience as a multimodal situational overview of the world, including the subject’s own body, which is considered one of the key functions of consciousness. All six theories describe neural mechanisms at different levels of organization, from the microlevel of (sub)cellular processes, through the mesolevel of intra-regional circuits, to the macrolevel of interactions between brain regions.

Overall, these studies tackle significant challenges in evaluating consciousness, including the following:

- Defining “Consciousness”: Achieving a precise definition of consciousness, which may only be fully clarified with the development of a universally accepted theory—a goal that remains unmet.
- Identifying Neural Correlates: Pinpointing the neural correlates of consciousness, including the minimal neural mechanisms sufficient for conscious perception and behavior.
- Determining Levels of Consciousness: Assessing various levels of consciousness, encompassing medical aspects of disorders in humans, potential manifestations in animals, and explorations in computer science and artificial intelligence.
- Exploring Cognitive Relationships: Investigating the relationship between consciousness and various cognitive brain functions, including attention, perception, thinking, decision-making, and behavior.
- Developing Distinct Criteria: Establishing criteria for distinct properties of consciousness applicable to psychiatry, neurophysiology, psychology, sociology, and other biomedical and social sciences.
- Assessing subjecthood and Qualia: Evaluating the degree of individual subjecthood in information processing and qualia (subjective sensory experiences) that shape perception of reality and subsequent behavior.
- Defining Consciousness and the Subconscious: Elucidating the relationship between consciousness and the subconscious, including the influence of acquired and instinctive behavioral programs, innate motivations, and their sublimations on conscious experience.

Gilbert Ryle’s The Concept of Mind (1949) challenges the Cartesian separation of body and mind by arguing that mental activity cannot be divorced from bodily functioning [39,40]. Ryle portrays consciousness—not only sensation and intellect but also self-reflection—as a capacity for observable actions, even while acknowledging a private, introspective dimension that remains inaccessible to external measurement. To clarify human cognition, he distinguishes between “knowing how” (procedural competence) and “knowing that” (propositional information), anticipating modern neurophysiological insights: rather than passively receiving sensory data, the brain actively constructs causal models that distinguish random from lawful events and then plans and evaluates outcomes. These subjective processes give rise to phenomenal consciousness or “what it is like” to perceive qualia, encompassing both contents of working memory and subconscious influences. Although Ryle’s emphasis on private mental life did not address social consciousness (e.g., Marx’s view that social being shapes consciousness), his core insight—that subjective experience manifests through action—foreshadows contemporary efforts to identify neural substrates of “knowing how” (e.g., motor planning circuits) versus “knowing that” (e.g., frontoparietal networks). In our review, we move beyond these philosophical distinctions to examine how evolutionary changes in neural architecture underlie both procedural and propositional aspects of subjectivity.

Recently, psychologists and sociologists have been developing and refining conceptual and empirical tools to study the personal and social nature of the self and identity, as well as the social, cultural, and historical formation and expression of the self. Perhaps the most comprehensive treatment of the interplay between the individual self and the multiple (collective) selves is found in the seminal volume edited by Ashmore and Jussim, Self and Identity: Fundamental Issues [41].

One direction of these studies is establishing a connection between subjective self-assessments and subjective well-being with belief in and the exercise of human free will as an activity aimed at achieving long-term, including socially significant, goals [42,43]. Free will is defined as the notion that decisions and actions should not be entirely determined by preceding causes that lie outside of our conscious control [44]. At the same time, unconscious brain activity may precede conscious causality and free will within the framework of the interrelation between the functions of consciousness and the evolutionarily older functions of the subconscious [45,46,47]. Contemporary neuroscience does not deny the existence of free will and moreover identifies certain neural and behavioral correlates of this consciousness-related phenomenon [47,48].

In turn, we consider it necessary to first address more fundamental questions, namely the following: (1) Why did consciousness emerge? (2) If consciousness is viewed as an integral functional system, what serves as its system-forming factor?

There is growing consensus that consciousness did not emerge suddenly in humans but evolved at certain stages of species evolution [49,50]. This is particularly true for animals with a sense of self, i.e., those that recognize their individuality and distinction from others, as demonstrated by the mirror mark test, successfully passed by dolphins, primates, and birds of the corvid family [51]. Some authors suggest that even certain species of fish may possess this ability [52,53]. Our framework builds on the neurobiological naturalism of Feinberg & Mallatt (2016–2022), who first identified the specific neural features that gave rise to proto-consciousness in early vertebrates and arthropods through weak emergence [13,14,15,16].

This review synthesizes empirical evidence for the evolutionary emergence of consciousness while acknowledging open questions about necessary/sufficient conditions. We trace neural and behavioral correlates across taxa without presuming inevitability. Throughout this article, terms such as “subconscious” are used in a strictly neurobiological sense, referring to functional systems of nonconscious information processing, as distinguished from psychoanalytic interpretations.

In light of the above, we propose a model of consciousness based on the following principles:

1. Natural Evolutionary Stage: The emergence of consciousness is a natural stage in the evolution of animals endowed with nervous systems, aimed at ensuring their survival in changing environmental conditions.
2. Subjectivity as Key Factor: The key systemic factor of consciousness is subjectivity, which involves the perception of the value of one’s existence and needs, and the distinction of the subjective “self” from the surrounding environment.
3. Subjective “Self” as Mediator: The subjective “self” acts as a “censor” for incoming conscious information and as a “conductor” for the implementation of complex behavioral programs aimed at satisfying the subject’s perceived needs.
4. Central Position of the “Self”: The subjective “self” occupies a central and distinct position in the subject’s worldview.

The purpose of this study is to develop an abstract model of consciousness based on subjectivity as the organizing principle, which emerged through the evolution of complex neurobiological information systems.

## 2. Consciousness as a Lawful Product of the Evolution of Information Systems

Since no universally accepted formalizations of the fundamental properties of consciousness, its origins, or its essence currently exist, we begin our narrative with a definition of consciousness.

### 2.1. Definition of Consciousness

Drawing from both biological and phenomenological perspectives, we define consciousness as an emergent phenomenon of subjective experience that organizes perception, memory, decision-making, and behavior into a coherent, goal-directed process. By “subjective”, we refer to the first-person qualitative experience of phenomena; by “phenomenon of experience”, we acknowledge that it arises from integrated neural (or potentially artificial) processes without being reducible to any single component of these processes.

The functional core of this subjective experience is the formation of the subject’s “self”, which establishes its distinct existence from the external environment, evaluates the priorities of its needs, and possesses the volitional capacity to actively pursue them. Another fundamental prerequisite for the emergence of consciousness is the subject’s ability to develop representations of the surrounding world based on acquired experience and causal reasoning. In this sense, consciousness serves as an organizer, censor, and regulator of behavioral programs acquired through life experience, ensuring the subject’s adaptation to environmental conditions and the fulfillment of its needs.

Consciousness operates as a functional system that processes vast amounts of information [35]. This necessitates a precise conceptualization of widely used but often ambiguously defined terms such as system, information, and information system.

### 2.2. Specification of Definitions: System, Information, and Information System

#### 2.2.1. Systematicity

The fundamental characteristics of any system include a set of interrelated elements that can be distinguished from their environment and the irreducibility of the system’s properties to the mere sum of its components. A more detailed exploration of this issue can be found in works on General Systems Theory [54,55,56,57]. In this context, it is important to highlight that the systemic approach—including system analysis—constitutes a core component of scientific methodology [58,59,60]. Such an approach involves verifying a specific system model, determining its typological classification, identifying causal relationships among subsystems, establishing connections between the system and other elements within its supersystem, and evaluating the fundamental principles and functional processes that sustain the system’s integrity.

From a systems perspective, biological organisms can be viewed as biological information systems (BISs) or functional systems (FSs), whose modules—sensory inputs, internal matrices of needs, memory and effectors—closely parallel those in engineered information systems. In BIS, “information” denotes intrinsic, organism-relative data rather than abstract Shannon signals, and the same organizing principles—feedback loops, negentropy and information processing—apply across both biological and artificial domains [61,62,63].

Currently, systemic methodologies are also applied to the study of mental processes and psychiatric disorders [61,62]. However, it is essential to recognize that all theoretical models, classifications, and philosophical definitions—while useful for generating hypotheses—remain subjective reflections of reality and must be evaluated empirically for plausibility.

#### 2.2.2. Information and Information Systems

The concept of information was rigorously formalized by Claude Shannon, who defined it as a reduction in uncertainty and showed that any communication system’s capacity can be quantified mathematically [63]. While contemporary information theory often takes the form of algorithms used in artificial intelligence [64,65,66]—and such algorithms can, in principle, characterize aspects of human cognition [67,68,69]—we adopt a broader, systems-level definition.

In this view, “information” comprises all recorded data about an organism’s internal and external environment that serve its functional needs. Biological ISs exhibit several defining properties:

Autonomy and Self-Organization: Cells and tissues maintain distinct boundaries from inert matter by relying on internal informational matrices (e.g., genetic and epigenetic codes) that regulate their states and needs.

- Interaction and Adaptation: Subsystems exchange signals—chemical, electrical, or mechanical—to coordinate growth, metabolism, and behavior. This exchange enables homeostatic regulation and responsive adaptation when conditions change.
- Negentropy: By harnessing energy (e.g., metabolic fluxes), living ISs counteract entropy, preserving and increasing structural complexity over time [70,71,72,73,74,75].
- Evolutionary Progression: Through mutation, selection, and developmental programs, biological ISs undergo gradual, irreversible transformations that enhance information processing capacity.

**Figure 1:**
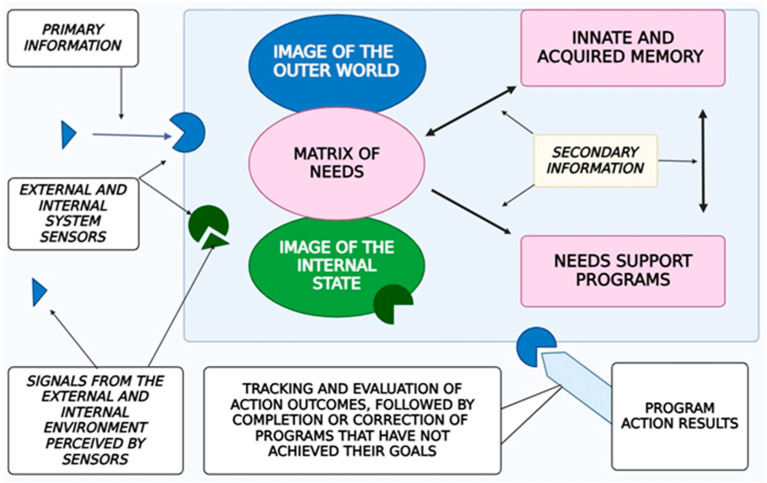
Conceptual model of biological information systems. Note: The system-forming component of an information system (BIS) is the matrix of needs and internal states, which the system strives to maintain while interacting with its external environment. Based on this matrix, programs are developed to process primary information, which is perceived by the system’s sensory structures. This primary information is then focused and integrated, leading to the formation of secondary (processed) information, which serves as the foundation for behavioral programs aimed at fulfilling the system’s needs. For communication with other systems within a broader supersystem, information systems exchange tertiary signal information, which is embedded in behavioral programs. Through feedback mechanisms, BISs continuously monitor the outcomes of their programmed actions, allowing for adjustments to these behavioral programs and, in some cases, modifications to the evaluation matrix of their internal state.

Figure 1 depicts a generic BIS model: primary signals (raw sensory inputs) are filtered according to internal needs and converted into secondary signals (refined representations) that trigger behavioral programs. On occasion, secondary information is stored in memory circuits, which later inform decision-making. Finally, tertiary signals—such as communicative sounds or pheromones—are transmitted externally to coordinate with other organisms or to modify the environment. In the context of human consciousness, secondary information corresponds to neural codes instantiated in working memory and attentional circuits, while tertiary information includes language, symbolic artifacts, and other means of transmitting abstract representations. This framework highlights both the shared regularities of ISs and the distinctive features of consciousness as a high-order biological information system.

Already in unicellular microorganisms—bacteria and other prokaryotes—complex BISs are evident. These BISs comprise internal and external sensory structures (e.g., receptors and allosteric enzyme centers), elaborate signaling pathways linking those sensors to the genome, and diverse regulatory proteins [76,77,78,79,80]. Together, these pathways form modular networks that process distinct classes of sensory inputs and generate appropriate adaptive outputs. Each module can be conceptualized as comprising three subsystems—receptor, transducer, and effector—that coordinate to maintain cellular function [81].

Like human cells, many prokaryotes deploy sophisticated stress response mechanisms to counter adverse conditions and navigate their environment via flagellar or gliding motility [82,83,84,85]. Similarly, numerous plant and animal cells employ pattern-recognition receptors to detect molecular signatures of danger, preemptively triggering protective responses [86,87]. In prokaryotes, the CRISPR–Cas system provides a form of acquired genomic memory, recording viral sequences to guide subsequent defense [88,89]. Microbial communities also generate tertiary information: they communicate through quorum-sensing molecules and can even launch informational “attacks” on other cells [90,91,92,93], while biofilm formation illustrates how bacteria engage in complex, networked IS interactions [94,95].

A fundamental BIS model underlies adaptive processes throughout biology. Anokhin’s theory of functional systems, for example, describes how afferent synthesis, motivation, memory, decision-making, action, and outcome evaluation integrate into coherent behavioral acts aimed at preserving homeostasis [35,96]. Thus, higher nervous activity inherits these core BIS principles—namely, a “needs code”, perception and appraisal of incoming signals, program generation to address those needs, networked information exchange, and the capacity for acquired memory.

Consciousness can be viewed as a high-level regulatory BIS emerging from neural structures. Its primary role is to coordinate neuromuscular programs, thereby enabling flexible adaptation to environmental changes. In contrast, homeostatic regulation (e.g., via the autonomic nervous system) operates largely independently of conscious control.

In animals with highly developed central nervous systems (CNSs), behavioral complexity provides suggestive but not definitive evidence for biological consciousness [30]. Behavioral complexity—particularly social and group behaviors—became evolutionarily advantageous in such species [97,98,99,100,101,102,103]. When behavioral programs conflict, consciousness functions as an arbitrator, integrating competing motivations. Although sustaining a large brain incurs high metabolic costs [104], the cognitive benefits often justify this expense by enhancing survival and reproductive success.

These observations raise two fundamental questions: Is consciousness an inevitable stage in the evolution of species? More broadly, might consciousness naturally emerge in any sufficiently complex BIS, including future autonomous technological systems? While definitive answers remain elusive, comparative data on convergent features of biological consciousness across diverse taxa may illuminate the factors that drive its emergence. Central to this inquiry is identifying the system-forming factor of consciousness—that is, the organizing principle responsible for its origin and principal phenomena. In the following sections, we will explore this question in depth.

## 3. Subjectivity as the Organizing Factor of Consciousness and Thought

### 3.1. Essential Conditions for the Emergence of Consciousness

The emergence of consciousness requires several biological prerequisites. First, brain volume and structural organization must be sufficient to support both working (operational) and long-term memory stores. Working memory maintains a coherent, moment-to-moment perception of the environment, with the subjective “self” (an organism’s sense of identity) at its center. Long-term memory is encoded in subconscious circuits and is retrieved into conscious awareness as needed.

Second, multiple brain regions must operate as an integrated network. Extensive reciprocal connectivity—particularly among sensory, associative, and limbic areas—ensures that incoming inputs, internal needs, and stored representations converge into a unified state. Motivational centers (e.g., hypothalamic and limbic nuclei) interpret physiological and environmental signals, informing the organism of positive or negative valence and thereby shaping goal states. In parallel, affective circuits generate positive and negative emotional signals that calibrate the degree of need satisfaction.

Third, thought processes—mediated by distributed prefrontal and parietal networks—construct causal models of the world. These models underpin the formulation of complex behavioral programs, enabling the organism to predict outcomes and select adaptive actions. When multiple motivational drives or behavioral strategies conflict, a central integrator (“arbitrator”)—embodied by the subjective “self”—resolves these conflicts by allocating attentional and executive resources.

Finally, vertebrate brains evolved the capacity to override instinctual reflexes in favor of higher-level behavioral programs because this flexibility conferred a selective advantage in unpredictable environments. Such overrides incur significant metabolic costs—especially the energy required to sustain a large, integrative cortex—but the resulting gains in adaptive decision-making and survival justify these expenditures.

### 3.2. Fundamental Model of Human Consciousness

**Figure 2:**
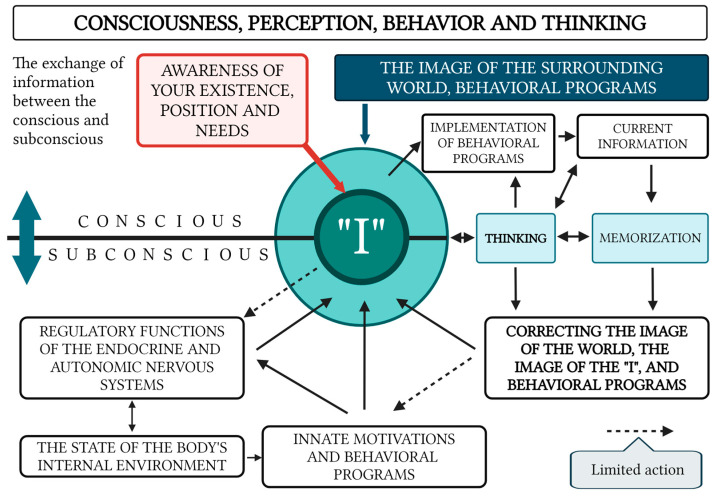
Basic model of human consciousness and its main functions. Note: In this figure, the term “subconscious” refers to neurogenically encoded memory patterns (innate and acquired) that are not currently present in conscious awareness but influence cognition and behavior. This usage is grounded in systems neuroscience and is distinct from psychoanalytic interpretations. Human consciousness is a complex system in which the presence of a subjective “I” serves as the system-forming factor. The foundation of consciousness is the mental representation of the surrounding world, maintained in working memory, with the self-positioned centrally within this representation. This image is constructed based on two primary sources: accumulated life experience and acquired memory on one hand, and real-time sensory input on the other. Within consciousness, continuous processes of analysis and synthesis take place, establishing causal relationships not only between sensory-perceived events but also with information retrieved from long-term memory storage. The innate instincts serve as the initial matrix for the formation of consciousness and the closely related process of thinking. However, in humans, these instincts undergo significant sublimation as part of social upbringing. On the basis of the constructed world model, with arbitration from the subjective “I”, complex multi-stage behavioral programs are developed and monitored for execution. Additionally, this world model incorporates an assessment of the body’s internal state. However, the influence of consciousness on the autonomic nervous system and humoral regulators of homeostasis is limited (represented by dashed arrows).

The fundamental properties of human consciousness are depicted in Figure 1, consisting of the following key components:

- The Subjective “Self”: This functions as the central organizing factor of consciousness.
- A Holistic Image of the External World and the Subject’s Own Body: Consciousness incorporates a comprehensive representation of the surrounding environment and the current state of the organism. With that, the image of the surrounding world is not simply a reflection in consciousness (a “photograph” of the reality surrounding the subject) but is generally a complex system with constantly established cause-and-effect relationships between individual phenomena within this dynamically changing system.
- Thought Processes: These enable the formulation of behavioral programs that are aligned with conscious needs and goals.
- Filtering and Organizing Sensory Information: Consciousness must systematize and filter the sensory input it receives in order to make it usable for higher-order processing.
- Integration of Information Through Thought: Thought processes integrate incoming data to form a cohesive understanding that drives behavior.
- Influence of Internal Bodily Changes: Consciousness is influenced by hormonal fluctuations, the autonomic nervous system, and motivational centers, although it has limited direct control over these processes (Figure 2).

Consciousness and the subconscious together form a unified, interconnected system characterized by constant information exchange. In this model, we use the term “subconscious” not in the classical Freudian sense, but as a functional neurobiological construct. It encompasses a set of innate and acquired memory programs that are not currently accessible to conscious awareness but can influence conscious processing or become consciously accessible later. This interpretation aligns with contemporary cognitive neuroscience and functional systems theory, as well as with recent formulations of precognitive processing [13,14,15,25,35].

**Figure 3:**
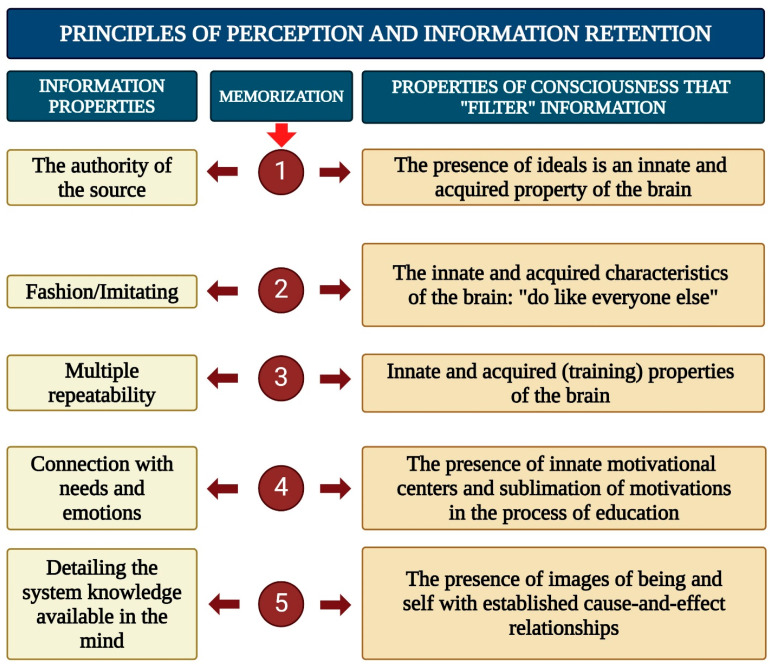
The five main “filters” associated with consciousness for encoding sensory information flowing into the brain. Note: Filtering and memorizing information that enters the brain is a stage of cognition aimed at distinguishing general from specific, cause from effect, primary from secondary, temporary from permanent, etc. For more detailed information about these filters that process incoming information streams into the brain, see the following references: [109,110,111,112,113,114,115,116,117,118]. The functional filters illustrated in Figure 2, through which information enters the brain, are determined by both innate and acquired properties of consciousness. A positive feedback loop is formed between these functional filters and the perception, cognition, and subjective image of the world that develop from early childhood. As a result, individuals tend to absorb information that aligns with these cognitive stereotypes. Ultimately, this phenomenon significantly influences the stable division of people into group types with distinct cognitive stereotypes. However, every individual has the ability to adjust these stereotypes and, accordingly, their informational filters through the accumulation of life experience.

The flow of information entering consciousness from the external environment must pass through several “filters”, as illustrated in Figure 2. According to some authors, subjective experiences such as memories and voluntary actions arise from the activity of interconnected neural generators in the brain’s cortex [105]. Furthermore, the formation of certain mental patterns is laid down during the first few years of life, and these patterns are subsequently adjusted throughout one’s lifetime [106]. In turn, universal models of thinking, behavior, and cognition, genetically programmed into the subconscious, manifest themselves as various archetypes that influence both individual and collective consciousness [107,108] (Figure 3).

Throughout life, the self (SFF) integrates bodily inputs, social roles, and future goals. In neurological terms, interactions among prefrontal, insular, and limbic circuits continuously refine one’s momentary “I” [118,119,120,121,122,123,124,125,126,127,128,129,130,131].

### 3.3. Morphofunctional Foundations of Consciousness

Consciousness is likely not selectively associated with specific brain structures but rather emerges as an integrative function of the brain as a whole. The human brain weighs approximately 1300–1400 g and is an extremely complex system, comprising around 10^9^ distinct types of neurons, approximately 10^14^ synaptic inter-neuronal connections, and a diverse repertoire of synaptic neurotransmitters and their receptors [118,119]. The complexity of the brain’s structure is determined not only by the vast number of diverse neuron types and extensive synaptic connections but also by the wide variety of molecular neurotransmitters and an even greater diversity of their receptors, which facilitate trans-neuronal information transmission [120].

In humans, the cerebral cortex accounts for about 82% of the brain’s volume but contains only 19% of its neurons. This discrepancy arises because the cortex is predominantly composed of white matter, consisting of highly branched and interconnected neuronal processes, including axons (signal transmitters) and dendrites (signal receivers) [121]. Despite constituting only about 2% of body mass, the brain at rest consumes approximately 25% of the body’s energy [122], underscoring the high metabolic cost of sustaining consciousness. However, as already noted, the presence of consciousness provides organisms with a more effective means of adapting to their environment.

**Figure 4:**
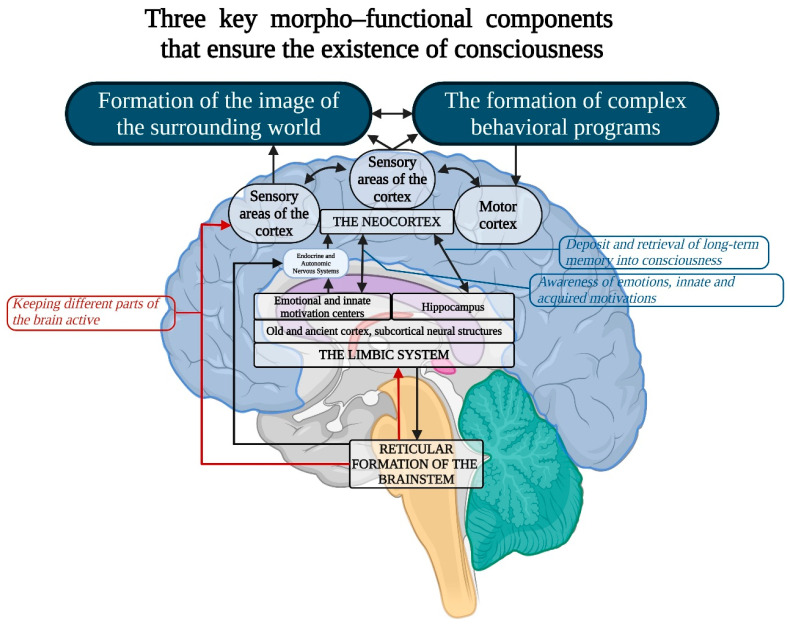
Consciousness as an integral function of various regions of the brain. Note. Consciousness is the functional product of the integration of various brain regions. This integration occurs vertically—from subcortical structures (limbic system, follicular formation), to the old and ancient cortex (limbic system, olfactory cortex), to the neocortex (sensory, motor, and associative cortical areas)—as well as horizontally, primarily between different cortical areas via long-range neuronal projections in the white matter. Extensive associative regions of the parietal and temporal cortex, as well as the prefrontal cortex (PFC), are primarily responsible for integrating and systematizing incoming information, storing long-term memory, and forming complex behavioral programs (especially in the frontal PFC). The sensory, motor, and associative cortical regions in Figure 4 are represented schematically and do not precisely match their true localization. For example, the visual cortex is situated in the occipital lobe, the auditory cortex in a specific part of the temporal lobe, the gustatory cortex in the lower anterior portion of the parietal lobe, the somatosensory cortex in the anterior part of the parietal lobe (postcentral gyrus), and the motor cortex in the posterior frontal lobe (precentral gyrus).

There are several primary morphofunctional components of the brain whose interaction is essential for the existence of human consciousness (Figure 4).

1. Neocortex: Throughout mammalian evolution, distinct cortical regions have expanded at varying rates, leading to a complex mosaic of specialized areas within the neocortex [123]. A prime example is the prefrontal cortex (PFC), crucial for planning complex cognitive behaviors and showing significant evolutionary divergence across species. In rodents, the PFC is predominantly agranular, lacking the prominent layer 4 (granular layer) that defines the granular PFC in primates [124,125]. In humans, the granular PFC expanded further, not only increasing in size but also enhancing connectivity with other association areas. This expansion facilitated abilities such as abstract reasoning, language, and sophisticated social behaviors [126].

Moreover, the neocortex plays a crucial role in both working memory and long-term memory storage. The process of encoding and retrieving long-term memories in the neocortex is heavily dependent on interactions with the limbic system, highlighting the complex interplay between different brain regions in supporting cognitive functions [126].

Key properties of human consciousness include prospective thinking (representation of the future), recollection of the past, understanding others’ perspectives (theory of mind), and spatial navigation. These functions are specific examples of the broader process of “self-projection”, which involves medial temporal, parietal, and frontal cortical networks. Notably, these regions exhibit high metabolic activity during passive rest and are collectively known as the default-mode network (DMN) [127]. The DMN plays a significant role in internally directed thought processes, further highlighting the complexity of human cognitive abilities. While rodents are valuable models for understanding basic neural processes, their lack of granular PFC regions limits their applicability in studying human-specific cognitive functions [128,129]. As brain size increased in humans, the development of new cortical areas was accompanied by modifications in connectivity, allowing for more sophisticated integrative functions [130,131].

Despite the fact that consciousness is an integral function of the brain, the role of individual neurons in the cortex in representing specific phenomena can be exclusive. For instance, individual neurons in the human medial temporal lobe are selectively activated by images of faces, animals, objects, or scenes. These neurons respond to entirely different images of specific people, landmarks, or objects, and in some cases, even to letter sequences representing their names [132].

**Figure 5:**
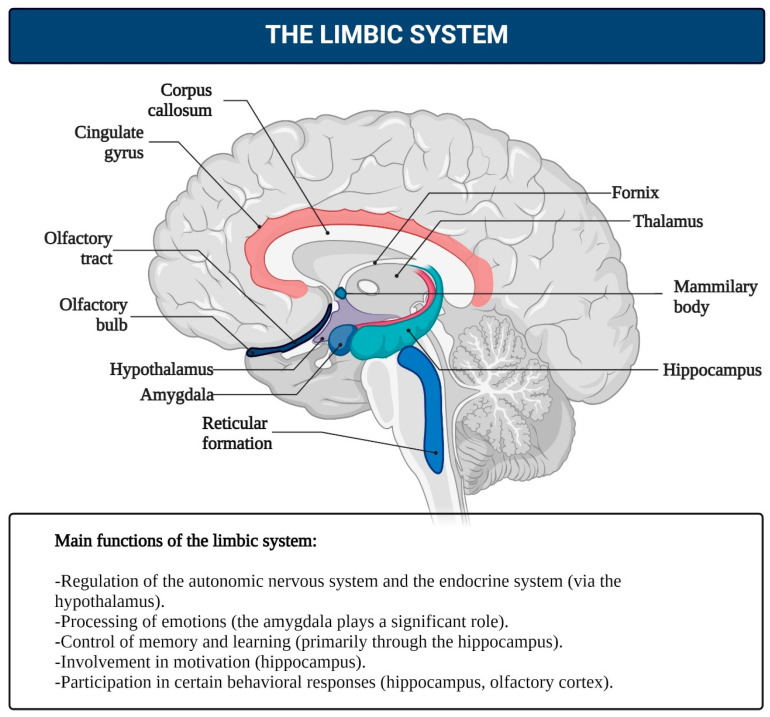
Key structures of the limbic system.

**Figure 6:**
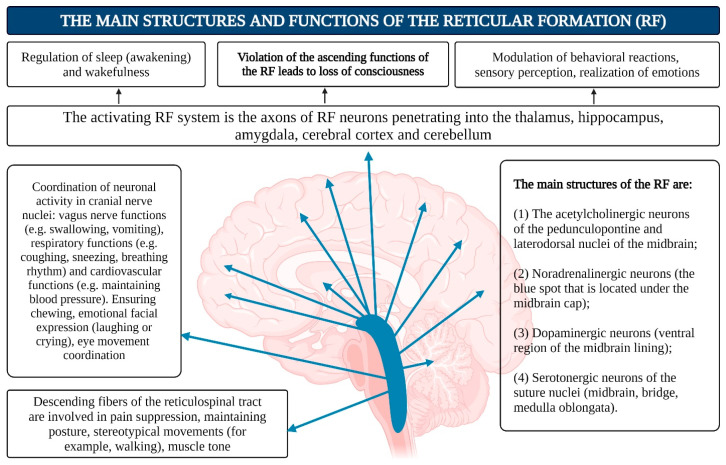
General characteristics of the reticular formation.

2. Von Economo Neurons (VENs)—large, spindle-shaped neurons of the fifth cortical layer: While consciousness is an integrative brain function, certain neuronal mechanisms may hold special significance in its realization. One example is von Economo neurons (VENs), large, spindle-shaped neurons located in the anterior insular cortex and anterior cingulate cortex (Figure 5), which play critical roles in self-awareness and the social facets of consciousness. These neurons are primarily found in humans but are also present in great apes and highly intelligent marine mammals [133,134]. Researchers agree on the selective vulnerability of VENs in certain pathologies where social behavior and emotional capacities are impaired, such as neuropsychiatric disorders including schizophrenia [135,136]. It is hypothesized that VENs help direct neural signals from deep cortical areas to more distant parts of the brain, integrating the neocortex with older cortical and subcortical structures of the limbic system [137]. In humans, strong emotions activate the anterior cingulate cortex, which relays signals from the amygdala—the primary center for emotional processing—to the frontal cortex. This system may function like a lens, focusing complex neural signals to facilitate emotional and cognitive processing.
3. Limbic System: This system functions as the center for emotions, as well as the organization of behavior, memory, sleep and wakefulness, stress response control, attention, and innate behavioral programs, including the execution of sexual instincts, feeding, aggression, and parental care [126,137,138,139,140]. The limbic system includes regions of the evolutionarily older cortex and subcortical (basal) nuclei, such as the olfactory bulb, anterior perforated substance, cingulate gyrus, parahippocampal gyrus, dentate gyrus, hippocampus, amygdala, hypothalamus, mammillary bodies, and reticular formation (Figure 5). The main functions of the limbic system are to regulate the autonomic nervous and endocrine systems (via the hypothalamus), process emotions (with a significant role played by the amygdala), and manage memory and learning (primarily through the hippocampus). It is also involved in motivation and some behavioral responses, including the olfactory cortex in humans. It is hypothesized that in the early stages of animal evolution, complex reflex mechanisms in the limbic compartments of the basal brain, which serve metabolic and other homeostatic processes, along with elements of the reticular activating system, contributed to the emergence of consciousness as a primordial emotion signaling that the organism’s existence was under threat [141,142,143,144,145,146,147,148,149,150,151,152].
4. Reticular Formation (RF): The reticular formation consists of nuclei and network structures that extend along the entire axis of the brainstem. The midbrain RF is closely connected with the limbic system, and some authors consider them to form a unified limbic–reticular complex. Projections from the RF (dendrites and axons) extend into most other brain areas, particularly the neocortex and limbic system, maintaining their active state. The RF is also responsible for regulating sleep–wake cycles, brain rhythms, vital centers (such as respiration and blood pressure), and cranial nerve nuclei in the brainstem. Damage or hyperactivation of the RF results in loss of consciousness [142] (Figure 6).
5. The thalamus.

Currently, many researchers emphasize the crucial role of the thalamus—a paired brain structure located at the center of the cerebral hemispheres between the brainstem and the cortex (Figure 5)—in the organization of consciousness [143,144,145,146,147,148]. Structures homologous to the vertebrate thalamus likely appeared during evolution in the common ancestors of chordates and arthropod invertebrates approximately 550–600 million years ago [149]. Among higher vertebrates (amniotes)—reptiles, birds, and mammals—this brain region began to develop independently around 320–330 million years ago, coinciding with the existence of their last common ancestor [150].

The thalamus serves as a relay hub, connecting the cerebral cortex on one side with the cerebellum and basal ganglia on the other. Specifically, it acts as a filter for sensory information transmitted from the sense organs to the cerebral cortex (excluding olfaction). Thus, the thalamus can be regarded as a central hub that integrates nearly all sensory and motor information before relaying it to the cerebral hemispheres.

Beyond its role as a sensory and motor relay, the thalamus includes nuclei of the limbic system (with its anterior part directly connected to the hypothalamus) and nuclei of the reticular formation, which regulate cortical activation and inhibition. The thalamus participates in the integration of different cortical regions through cortico-thalamo-cortical and thalamo-cortico-thalamic circular connections. The widespread and diffuse nature of these thalamic projections suggests that the diffuse-projecting nuclei of the thalamus play a key role in regulating overall cortical and subcortical excitability, levels of consciousness, CNS arousal and activity, attention concentration, and the regulation of sleep–wake cycles.

Notably, thalamic activation has been shown to accelerate the return to consciousness in patients recovering from anesthesia and to improve the condition of patients in a coma. Additionally, several studies indicate that central thalamic stimulation at frequencies of 40–100 Hz induces transitions to conscious states in rodents, macaques, and humans [141,151,152].

The thalamus influences conscious states by modulating the integrative properties of the cerebral cortex, including oscillatory synchrony, neural resonance (responses to inputs at specific frequencies), functional connectivity, and the regulation of oscillatory synchrony [93,100]. During wakefulness, tonic bursts in the thalamus adjust the responsiveness of cortical networks to optimize the efficient transmission of information, thereby contributing to a responsive, functionally coherent conscious state [153]. It is also suggested that the thalamus may play a role in compressing multidimensional cortical information into a contextually rich but detail-limited space [141,154].

Furthermore, Aru J. and colleagues propose that large pyramidal neurons in layer 5 of the neocortex (L5p) play a strategic role in the organization of consciousness, as they are positioned at the intersection of cortico-cortical and thalamo-cortical loops in mammals [34,145]. According to these authors, L5p neurons function as cortical output units that integrate the activity of cortical columns and serve as critical switches of consciousness, ultimately enabling the convergence of different classes of information in a complex operation governed by the binding properties of L5p neurons.

### 3.4. Decortication Experiments in Rats

Decortication studies demonstrate that many basic behaviors persist despite extensive cortical loss. Rats with near-complete ablation of the neocortex continue to feed, groom, swim, navigate, and fight, and they maintain sleep–wake cycles and circadian motor rhythms—albeit often at inappropriate times or locations, reflecting impaired contextual regulation [153,154,155,156,157]. Hippocampal removal alone produces only minor deficits in innate behavior but prevents formation of new learned tasks, confirming its critical role in memory encoding and retrieval [154]. Remarkably, rats with over 95% of their neocortex removed still engage in reproductive behaviors, underscoring the resilience of instinctual programs [155].

Key observations from these experiments include that (1) cortical integrity is necessary for spatial and contextual learning, (2) brainstem and other subcortical circuits suffice to support simple cue–response associations and basic survival behaviors, and (3) while the cortex enhances flexibility and complex integration, it is not strictly required for rudimentary adaptive functions. Thus, even in the near-complete absence of cortex, subcortical circuits can support basic “proto-conscious” functions. From an evolutionary standpoint, these findings imply that the SFF (system-forming factor of consciousness) first emerged in subcortical architectures before being elaborated by neocortical expansions.

## 4. Evolutionary Aspects of Consciousness

In his seminal publication, Paul Cisek proposed a plausible theory of behavioral complexity in biological species, developed using the “phylogenetic refinement” method [158]. According to this method, behavioral programs gradually evolve from simple to complex, following the sequence of phylogenetic changes that have occurred throughout evolution. This theory suggests that the basic feedback control of interactions with the environment was established during vertebrate evolution, ultimately leading to the functional architecture of the mammalian brain.

Behavior, as described in this framework, consists of perceptual (subjective representation of reality), cognitive, and action-oriented (goal-directed) systems, each of which can be further differentiated. The neural correlates of presumably unified functions, such as “working memory” or “decision-making”, appear to be distributed throughout the brain. In contrast, other correlates, such as “attention” and “intention”, are concentrated in specific brain structures and often overlap even at the level of individual cells. The core idea of Cisek’s approach is the gradual refinement of behavioral theories, wherein each hypothetical mechanism is considered an extension of an ancestral one. He emphasizes the presence of conflicting behavioral programs (e.g., approach and avoidance), necessitating complex regulatory mechanisms, including feedback-based control systems.

In this regard, we propose that the emergence of subjectivity—as a necessary prerequisite for consciousness—became an inevitable evolutionary response to the increasing complexity and contradictions of behavioral programs based on acquired memory. These contradictions drove the need to develop sophisticated cognitive frameworks for solving vital problems. We argue that the emergence and evolution of consciousness, as a synthesizer and “arbitrator” of these cognitive frameworks, is not strictly linear along the vertical trajectory of the evolutionary “tree”. Instead, behavioral complexity can also develop horizontally, as exemplified by the evolution of invertebrates, particularly cephalopod mollusks.

A comparative model of the neural architectures of cognitively capable cephalopods and vertebrates suggests that the cephalopod brainstem is functionally analogous to the forebrain and midbrain of vertebrates, while the pedal and pallio-visceral lobes of the cephalopod brain correspond to the spinal cord and hindbrain in vertebrates [159]. However, this case primarily represents evolutionary convergence (the convergence of functionally similar phenomena with distinct evolutionary and genetic origins). The common ancestor of all bilaterian animals (including vertebrates and mollusks) likely already possessed a chain or strand of interconnected neural cells and a primitive central nervous system, which underwent fundamental modifications along different evolutionary trajectories [160].

There is increasing evidence suggesting that certain signs of consciousness are present in animals, including emotional behavior, cognitive abilities, acquired memory, and various forms of individual character and behavior, as well as the presence of will in achieving goals. From an evolutionary perspective, different forms of consciousness can be conditionally divided into three categories:

1. Preconsciousness: This category refers to instances where the existence of consciousness as a unified phenomenon in certain animal species cannot be confirmed or denied with sufficient certainty. In humans, manifestations of preconsciousness are characterized by vague, subconscious memories that do not form into clear recollections [161]. In animals, verification of preconsciousness may be associated with insufficient scientific data to confirm the presence of fully developed subjective consciousness, as well as with the marginal state of their higher nervous activity.
2. Biological (animal) consciousness: This form of consciousness is defined by the presence of a sufficient number of identifiable traits that allow its verification. Achieving absolute consensus among all specialists on this issue is an inherently unattainable goal.
3. Culturally mediated or socialized human consciousness: This form of consciousness is shaped and influenced by the cultural environment of society.

Moreover, there is currently no strong reason to categorically dismiss the potential emergence of nonbiological, artificial consciousness, for example, in computer systems.

### 4.1. The Origins of Consciousness or Preconsciousness in Animals

#### 4.1.1. Some General Characteristics of Nervous System Evolution

Certain indirect signs of consciousness can be identified in various animal species. Meanwhile, it becomes necessary to at least approximately evaluate the stages in animal evolution where prerequisites and conditions for the emergence of various forms of biological consciousness first appeared, starting from invertebrates and including a comparative analysis of invertebrates and vertebrates.

The appearance of all modern animal phyla (up to 35) is linked to the Cambrian “evolutionary explosion”, which occurred approximately 540–520 million years ago [162,163]. This event is initially associated, even before the Cambrian, with two alternative directions in invertebrate evolution: (1) the formation of protostomes and (2) deuterostomes. From these, we can identify at least three main evolutionary directions that are, in our view, most relevant to nervous system development: (1) protostomes/arthropods (phylum)/insects (class); (2) protostomes/mollusks (phylum)/cephalopods (class)/octopuses (order); and (3) deuterostomes/chordates (phylum)/fish-like jawless vertebrates (superclass)/jawed fish (multiple classes)/amphibians (class)/higher vertebrates (classes: reptiles/birds, and, in addition, independently, mammals).

The formation of vertebrates from ancestral invertebrate chordates is associated with the phenomenon of two whole-genome duplications [164]. All existing vertebrates share the first duplication, which occurred in the Cambrian period via autotetraploidization (a direct genome doubling). In contrast, the second duplication is found only in jawed vertebrates and occurred later through allotetraploidization, i.e., genome duplication following interspecies hybridization from two now-extinct ancestral species [165]. All modern vertebrates possess characteristic brain regions, including a forebrain, as well as a hierarchical neuroendocrine system [166,167,168].

Additionally, vertebrates not only have a closed circulatory system (which is also present in some invertebrates) but also a complexly regulated microcirculatory blood system with continuous and differentiated endothelial lining of microvessels, including contractile arterioles and pre-capillaries, capillaries, and post-capillaries [168]. Unlike invertebrates, all vertebrates also possess a complex adaptive (acquired) immune system, based on the integrative function of various lymphoid organs [168,169]. These three systems in vertebrates are in synergistic evolutionary interconnection and mutually regulate each other’s functions in each specific organism.

In particular, the most advanced classes of vertebrates (birds and mammals) are characterized by homeothermy (warm-bloodedness), which is regulated by the CNS and the microvascular network, while, in turn, constant body temperature determines CNS stability [170]. The high overall organization level of vertebrates, including the CNS, allows for the formation of a complex brain-associated functional system of consciousness that controls animal behavior. However, evolutionary prerequisites for the formation of consciousness are also observed with varying degrees of expression in many invertebrate taxa. Evidence of these traits has been observed in several types of invertebrates. Evidence of these traits has been observed in several types of invertebrates, including arthropods, mollusks, echinoderms, and chordates [171,172,173,174,175,176,177]. In particular, invertebrates possess not only complex innate behavioral programs but also the ability to modify them through learning and conditioned reflexes. For example, the presence of purchased experience, some cognitive abilities, and behaviors resembling emotions are characteristic of many insect species and other invertebrates [173,174,176]. However, invertebrates generally have relatively underdeveloped brains and a limited capacity for acquired memory. Unlike various classes of vertebrates, invertebrates lack brain structures homologous to the reticular formation and limbic system found in vertebrates [177,178].

#### 4.1.2. Insects

A characteristic feature of most insect species is their high ratio of adaptation efficiency to a relatively low level of overall species organization compared to vertebrates. This is primarily expressed in the exceptional diversity and complexity of innate behavioral programs in these animals, which, in certain insect species, include mechanisms for acquired neurogenic memory [179,180,181,182,183,184].

For example, bees encode foraging vectors using optic flow and polarized light signals—an impressive neural computation for an animal with roughly one million neurons [185,186,187,188]. They navigate effectively by integrating these cues into spatial representations, yet they lack the advanced telencephalic circuits (analogous to vertebrate hippocampal structures) required for explicit cause-and-effect modeling. Ants similarly demonstrate sophisticated spatial navigation and problem-solving behaviors, as well as complex social organization and division of labor at the colony level [189,190]. These collective activities suggest distributed processing across the colony, rather than individual, model-based reasoning.

Based on such data, insects appear to possess a limited form of working memory—drawing on long-term memories to represent environmental features and guide navigation. However, no convincing evidence currently indicates that they construct internal models based on systematic cause-and-effect relationships among external phenomena. Nevertheless, adult insect brains exhibit significant plastic and physiological changes in response to environmental variation, a distinguishing feature of this class of invertebrates [191,192].

Insects also possess an innate immune system capable of modulating neural function—for example, microbial infections can alter octopamine pathways and influence behavior [193,194,195]. However, they lack the integrated neuroendocrine–limbic axes found in vertebrates that underpin emotional arbitration. Their compartmentalized nervous system consists of several ganglia and a relatively limited neuron count, preventing the emergence of cortical-like associative areas [195]. Additionally, an open hemolymph circulation and primitive respiratory organs constrain oxygen transport, thereby limiting body size and complexity [196,197,198].

Despite their typically short lifespans, insects maintain high reproductive rates and rapid genetic variability, enabling efficient adaptation—including speciation—within changing environments [197,198,199,200,201].

In sum, insects exhibit a very high diversity of innate behavioral programs and only modest capacities for working and long-term acquired memory. While various perspectives debate the extent of insect cognitive abilities [202], current evidence does not support the presence of all conditions necessary for subjective consciousness. In contrast, certain invertebrate taxa—most notably cephalopod mollusks such as octopuses—show clearer neuroanatomical prerequisites for more advanced forms of subjectivity.

#### 4.1.3. Cephalopods, Octopuses

Cephalopods occupy a special place among invertebrates in terms of overall species organization and nervous system structure, particularly octopuses [203]. For instance, the circulatory system of octopuses includes three hearts (one primary heart) and a closed vascular system, including contractile vessels (arterioles) and exchange microvessels (capillaries), but without a continuous endothelial lining [203,204,205,206,207]. Unlike their simpler mollusk relatives, cephalopods have developed a complex nervous system that combines centralized and distributed control [14,159,208,209,210,211,212,213,214,215,216,217,218,219,220,221,222,223,224,225,226,227,228,229,230,231,232,233,234]. The nervous system of an octopus typically contains around 500 million neurons, four orders of magnitude more than most other mollusks and over two orders of magnitude more than the most advanced insects (for example, cockroaches and bees have only about one million neurons) [206]. The size of the nervous system in modern cephalopods, normalized to body weight, falls within the same range as vertebrate nervous systems—smaller than in birds and mammals but larger than in fish and reptiles [206].

Compared to vertebrates, octopuses have a less advanced immune system based on innate mechanisms [207,208]. However, this is one of the most complex immune systems among invertebrates. Thus, the degree of overall species organization and nervous system complexity in octopuses and other cephalopods allows them to be considered candidates for possessing some level of subjective consciousness.

Some authors suggest that cephalopods display behavioral parallels with birds and mammals despite the significant evolutionary distance [159,209,210,211]. For example, octopuses are thought to exhibit morphological and functional lateralization relative to their large brain, similar to vertebrates. They rely heavily on learning in response to visual and tactile stimuli, can form simple concepts, and demonstrate complex spatial orientation and memory of feeding locations from the recent past. Interestingly, the organization of cellular layers at the blood–brain interface in octopuses suggests that it may serve to limit permeability between blood and brain, similar to the blood–brain barrier in vertebrates [205,206]. Previously, the presence of a blood–brain barrier was considered an exclusive advantage of vertebrate nervous systems.

Octopuses, however, lack a spinal cord and a clear division of the nervous system into central and peripheral components, as most neurons are associated not with the brain but with the eight limbs [204]. Most of the planning, computation, and execution of stereotyped arm movements occurs within the nervous system of the arms themselves, which limits the synchronization of all eight limbs when working in unison. Unlike vertebrates, cephalopods do not have a somatotopic representation of their motor functions; instead, they rely on parallel, overlapping circuits in both the central and peripheral nervous systems [14,227,228]. This decentralized system provides an alternative pathway to cognition and possibly a unique type of consciousness [213,229].

Genomic studies have shown that cephalopods possess unique genetic features, including evolutionary expansions of gene families associated with nervous system development, which supports their cognitive abilities [230,231]. Additionally, the expanded functions of stem cells may be key to understanding the evolution of the large and complex brain in cephalopods, possibly through genetic strategies similar to those in mammals, such as increasing the number and diversity of neurogenic stem cells [232].

Recent studies have begun to explore the potential for self-awareness in cephalopods using modified mirror self-recognition tests. In these cases, some octopuses exhibit unusual movements, tactile exploration, or adaptive changes in behavior when in front of a mirror, which may suggest a certain degree of self-awareness [232]. However, interpretations of such behavior remain debated due to differences in sensory modalities and neural architecture between cephalopods and vertebrates. Further research is needed to determine whether these responses indicate true self-recognition or are imitation effects [232].

The “Community of Minds” hypothesis by Carls-Diamante (2017) proposes that octopus’s consciousness may not be unified but rather composed of semi-autonomous regions within the nervous system that operate independently [227]. These questions underscore the complexity of understanding consciousness in organisms with such different nervous systems as vertebrates and cephalopods and challenge the necessity of a single model of consciousness [229,233].

Additionally, certain other features of the cephalopod nervous system can be highlighted:

- Small Neurons: Cephalopods possess small neurons (3–5 μm in diameter), which allow for a higher density of neurons in the brain. This increased density may enhance the brain’s ability to process complex behavioral responses and cognitive abilities [212].
- Limited Somatotopy: Generally, cephalopods lack a somatotopic representation of their body in the brain, but recent data indicate somatotopy in the basal lobe of squid brains [233].
- Efferent Modulation of Sensory Receptors: The function of efferent modulation of sensory receptors allows cephalopods to modulate sensory information, which is closely related to attention and selective perception, contributing to the accumulation of acquired experience [234].
- Complex Neural Circuit Architecture: The intricate architecture of cephalopod neural circuits, particularly in the dorsal basal and subvertical lobes of the brain, is thought by some researchers to mirror certain cortical processes found in mammals, responsible for learning and memory, and may be functionally similar to the mammalian hippocampus [159,235,236].

These cephalopod capabilities likely evolved as adaptive responses to environmental pressures, supporting the argument that complex cognition—and, consequently, the phenomenon of consciousness—can arise independently along various evolutionary paths. Given the above, we consider it possible to define the state of higher nervous activity in octopuses and certain other cephalopods as preconsciousness. This, in turn, emphasizes the need for further investigation into individual phenomena and mechanisms of consciousness in cephalopods to more accurately characterize it, especially in relation to subjectivity as a system-forming factor of consciousness.

#### 4.1.4. Vertebrates

All vertebrates possess three primary structural divisions of the brain:

- The forebrain consists of the telencephalon and diencephalon. The telencephalon is the most cranial region of the central nervous system in humans, which matures into the cerebrum. The dorsal part of the telencephalon (the pallium) develops predominantly into the cerebral cortex in mammals, while the ventral telencephalon (the subpallium) forms the basal ganglia. Primitive cortical structures are already present in agnathans vertebrates, indicating that these structures likely arose in the last common ancestor of jawed and jawless vertebrates approximately 460 million years ago [236]. The diencephalon includes the thalamus, metathalamus, hypothalamus, epithalamus, prethalamus (or subthalamus), and the pretectum.
- The midbrain is located centrally beneath the cerebral cortex. It consists of the tectum, the tegmentum of the midbrain, the cerebral aqueduct, the cerebral peduncles, as well as several nuclei and bundles of nerve fibers.
- The hindbrain serves as the transition to the spinal cord. In humans, it includes the pons, cerebellum, and medulla oblongata.

Certain regions of the midbrain, diencephalon, and telencephalon are structurally and functionally interconnected, forming what can be considered a unique functional complex known as the limbic system [215]. This system is generally characterized by its direct involvement in processes aimed at ensuring the survival of both the individual and the species.

According to some authors, the first conscious sense in vertebrates may have been vision [159]. Vision, in combination with additional sensory systems, transformed primitive reflexive systems into a brain capable of generating images that represent both the external world and the internal state of the body. These authors argue that even the simplest vertebrates, such as lampreys, possess the minimal requirements for sensory consciousness and qualia

Behavioral data indicate that robust working memory and voluntary attention—minimal criteria for sensory consciousness—are present in mammals and birds but generally absent in amphibians [213]. In contrast, certain bony fish exhibit not only these basic capacities but also long-term acquired memory, suggesting rudimentary subjective experience. For example, zebrafish (Danio rerio) can recognize individual conspecifics after a 24 h delay, demonstrating long-term social memory previously documented only in mammals [214]. Danio rerio are widely used to assess cognitive functions (learning, memory, stress responses) and exhibit individual differences in traits such as boldness, exploratory behavior, and spatial orientation, which modulate learning and memory under predator threat [206,220].

Amphibians generally lack robust working memory and voluntary attention, whereas many bony fish and most reptiles (particularly certain lizards, turtles, and snakes) exhibit rudimentary elements of both—suggesting an early vertebrate substrate for sensory consciousness [221,222]. Reptiles, birds, and mammals (amniotes) share homeothermy and an advanced limbic–forebrain axis, enabling a progression from primary (anoetic) to knowledge-based (noetic) and self-reflective (autonoetic) consciousness. According to Grinde, early amniotes (circa 320 Ma) evolved consciousness primarily through the incorporation of affective signals as decision-making strategies [223]. Fabbro and colleagues further propose that vertebrate primary consciousness is supported by a subcortical system (brainstem, hypothalamus, central thalamic nuclei), while more complex noetic consciousness emerges from forebrain structures (medial and lateral cerebral hemispheres). Self-awareness (autonoetic consciousness) may extend beyond humans to primates and other mammals and birds possessing highly developed nervous systems and homeothermic physiology [224].

### 4.2. Biological Consciousness and Signs of Subjectivity in Animals

Despite our current understanding of the broad spectrum of human emotions, there is no consensus within the scientific community on how to define sensory emotions and other manifestations of consciousness and self-awareness in animals. In this case, researchers often rely on comparing possible manifestations of consciousness in animals with similar reactions in humans. Such comparisons can provide evidence of a certain biological level of consciousness and selfhood, particularly in highly mentally organized mammals and certain bird species. Even laboratory mice, which cannot be classified among the most intellectually advanced mammalian species, possess significant working memory capacity and utilize it to solve intellectual tasks [236,237].

#### 4.2.1. Selfhood in Animals

Self-awareness can be understood as a process in which an individual, human or animal, becomes the object of its own attention. Self-awareness is thought to occur at different levels, ranging from low to high complexity [238]. It appears that only social animals consistently demonstrate self-recognition, while solitary species studied thus far seem to lack this trait. For example, mirror self-recognition tests (including self-directed behaviors) have demonstrated self-awareness in chimpanzees (*Pan troglodytes*), orangutans (*Pongo pygmaeus*), bottlenose dolphins (*Tursiops truncatus*), bonobos (*Pan paniscus*), and cleaner wrasses (*Labroides dimidiatus*) [239]. All of these animals are highly social, except for adult male orangutans. It is suggested that social animals have more opportunities for cognitive development than solitary species, which typically engage with less complex physical challenges. Some members of the Corvidae family (crows and related species) have also passed mirror self-recognition tests [51]. It is likely that the registry of animals possessing self-awareness will continue to expand over time [240].

While the mirror mark test has provided valuable insights into self-recognition, its failure in any given species cannot be taken as definitive evidence against internal self-awareness. Sensory-modality biases, individual habituation and ecological factors often obscure genuine self-representation. For animals in which vision is not the primary sense, alternative assays—such as olfactory self-recognition tests in wolves and dogs—have revealed clear self-awareness when tailored to their dominant modality [241,242]. Conversely, the cleaner wrasse (*Labroides dimidiatus*), a lower vertebrate, has demonstrated all three classical mirror-test phases (social response to its reflection, contingency-checking behaviors and mirror-guided removal of a visible mark), underscoring that even fish can pass self-recognition tasks when experimental design and interpretation respect species-specific sensory and behavioral repertoires [53,243]. These findings argue for a pluralistic, multisensory approach to assessing self-awareness rather than sole reliance on visual mirror tests.

#### 4.2.2. Learned Animal Communication

Many experiments have shown that certain species, including chimpanzees, orangutans, capuchins, elephants, wolves, hyenas, keas, bottlenose dolphins, and red-fronted parrots, understand the need for a partner and cooperation, while others, such as otters, rooks, ravens, and African gray parrots, do not [244]. Recent studies have demonstrated that great apes can use communication, particularly visual signals, to facilitate cooperative tasks [245]. Additionally, for intraspecies communication, these primates, such as bonobos (Pan paniscus), can use whistling in combination with high-pitched calls, with unique characteristics specific to different populations of these animals [246]. However, this contrasts with humans, who can use vocal communication. In humans, the ability to speak is linked to two-point mutations in the FOXP2 transcription factor gene, which occurred after the phylogenetic split between chimpanzees and humans, approximately 8–5 million years ago [247]. The ability to imitate sounds among conspecifics is largely unique to humans among primates, though several bird and mammal taxa independently evolved this ability [248]. There is evidence of vocal learning in many bird species and some mammals, such as bats, elephants, and marine mammals. Furthermore, evidence of both vocal and gestural learning has been found in two bird species (budgerigars and African gray parrots) and cetaceans [239]. Similarly to humans and unlike great apes or wolves, domesticated dogs demonstrate gaze alternation between a human’s face and various objects from an early age, provided they are well-socialized [249]. It appears that they have a genetic predisposition to communicate with humans through gaze alternation in the early stages of ontogeny. However, close interaction with humans may be required for this ability to manifest, highlighting the interactive influence of domestication and environmental factors on the behavioral development of dogs.

In socialized animals, particularly mammals, brain evolution has occurred not only to solve individual problems but also to enable group interactions, where the subjectivity of individual animals manifests unevenly depending on their dominance or submissiveness. This is especially relevant for animals such as lions, wolves, hyenas, Arctic reindeer, elephants, and chimpanzees [250,251,252,253,254,255,256]. Additionally, there is currently no reason to deny that many species of songbirds and parrots are capable of social play behavior directly related to the complexity of their brain organization and cognitive abilities [257]. The largest and most intelligent representatives of songbirds belong to the family Corvidae, many species of which are characterized by complex socialized behavior [258].

At the same time, collective animal behavior is largely characterized by typical behavioral patterns, indicating a significant role of innate mechanisms in its manifestation. However, such comparative studies are generally conducted under relatively standardized environmental conditions for different populations of the same species. Meanwhile, collective behavior in animals, such as monkeys and bottlenose dolphins, can be more substantially influenced by acquired features of intra-group communication in different populations of these species living in diverse environmental conditions [256,259]. Moreover, the convergent use of synchrony in group behavior among individual animals is a common socialized adaptation mechanism observed in both humans and bottlenose dolphins [260].

However, it would be incorrect to draw direct parallels between socialized behavior in humans and animals, as humans are profoundly influenced by the civilizational-cultural environment of supra-biological level social systems. Simultaneously, we believe that the social behavior of modern humans has evolutionary roots that extend deeply into the biological consciousness of ancestral animal species, as well as directly ancestral representatives of the Homo genus to Homo sapiens.

#### 4.2.3. Creativity and Flexible Thinking in Animals

Experimental studies show that primates and birds possess a range of creative abilities, including innovation in foraging, social learning, and tool use [261,262,263,264]. A series of experiments has shown that orcas (*Orcinus orca*), as top ocean predators, demonstrate several complex cognitive abilities, particularly flexible thinking or creativity when hunting for food [233]. Among primates, differences in intelligence and artistic abilities correlate best with differences in the number of cortical neurons and synapses, as well as information processing speed [79]. One manifestation of self-awareness and higher-order cognitive functions is the acquired skill of intentional deception to manipulate the behavior of group members, as seen in certain species of cetaceans and primates [264,265,266,267]. Corvids exhibit intellectual behaviors previously attributed only to primates [268]. Surprisingly, despite their relatively “primitive” neural architecture, corvids have evolved to the level of primates in terms of comprehensive concept learning and, in some tests, have even surpassed them. This suggests the convergent importance of abstract concept learning for species survival [269]. Crows create tools from leaves, use them to obtain food, and pass this knowledge to other crows through social learning; magpies develop an understanding of object permanence and can use this skill to the same extent as humans; and jays are capable of recalling events that occurred at a specific time or place, which was once considered unique to humans [270].

The ability of animals to learn abstract symbols is a key aspect of higher cognition. Learning abstract concepts, such as “same/different” and “matching to sample”, provides the foundation for many other forms of “higher” cognition [271]. Experiments have shown that chimpanzees can represent both the quantitative and ordinal aspects of numbers using Arabic numerals to some extent [272]. A comparative analysis of children aged 3 to 5 years, chimpanzees, and capuchin monkeys in a unified test based on the concept of “superordinate hypotheses” demonstrated that children, and to a lesser degree chimpanzees, exhibited the ability to perceive and use abstract patterns. In contrast, capuchin monkeys did not provide convincing evidence of forming abstract knowledge in solving tasks in this test [273]. However, capuchin monkeys are capable of solving spatial tasks with objects within the framework of spatial relational thinking models [274]. Like humans, monkeys seem to evaluate combinations of objects conditionally. Additionally, both humans and monkeys appear to use similar neural substrates for assessing the common characteristics of individual objects, such as the orbitofrontal cortex [275]. It is known that neurons in the prefrontal cortex encode rules, goals, and other abstract information, which are believed to underlie cognitive, emotional, and behavioral flexibility. However, observations in humans and experiments with monkeys show that the amygdala, a brain region traditionally considered an emotion mediator, also encodes abstract information, which may support this flexibility [276].

Moreover, animals that are less intellectually advanced than monkeys are also capable of learning abstract concepts. For example, perch-like angelfish from the family Pomacanthidae demonstrate an understanding of the abstract concept of “less/more” [277]. Pigeons can perform two-element conditional categorization of “same/different” with high accuracy [278]. Rats are capable of extracting and generalizing information from a given training situation to a new one [279]. This ability underlies abstraction, which is a hallmark of human cognition and essential for complex information processing.

#### 4.2.4. Sensory Emotions in Animals

Sensitivity in animals—the ability to consciously experience affective states—is widely recognized in ethics and law as the foundation of our obligations to protect animals [280]. Anatomical pathways of subcortical systems responsible for generating emotions originate in the ancient medial regions of the midbrain and diencephalon, as well as the subcortical basal nuclei of the telencephalon, which are preserved in all mammalian species [281]. The activation of these systems is subjectively experienced and manifests as “reward” and “punishment” effects, thereby facilitating learning and memory (secondary processes), as well as thinking, reflection, and other higher mental abilities (tertiary processes). Many highly organized mammalian species, like humans, can distinguish vocal expressions of emotion. Vocalizations can influence the affective states of receivers through direct (startle reflex) or indirect effects (affective learning), leading to emotional contagion within a group of interacting animals [282]. Moreover, dogs, horses, and to a lesser extent cats, demonstrate functional understanding of human emotional signals and adjust their behavior according to the valence and intensity of the emotional message conveyed [283,284,285,286]. Birds, too, are capable of experiencing both negative and positive emotional states. Research shows that birds exhibit complex emotional responses, similar to those of mammals, including facial and bodily expressions of fear and frustration [287,288,289]. At present, various mammalian species, including rodents, have been shown to exhibit emotional empathy toward members of their own species [280,289,290,291,292]. It is likely that in some of these mammals (primarily cetaceans and higher primates), primary emotional empathy may interact with higher cognitive functions, giving rise to feelings such as compassion or sympathy [281,291,292]. Certain bird species, particularly those from the corvid family, are also capable of empathy. For example, crows respond to the emotional needs of other crows and express empathy through prosocial, helping behavior toward valued partners [293].

#### 4.2.5. Differentiated Roles of Cortical and Noncortical Structures in Bird and Mammal Consciousness

**Figure 7:**
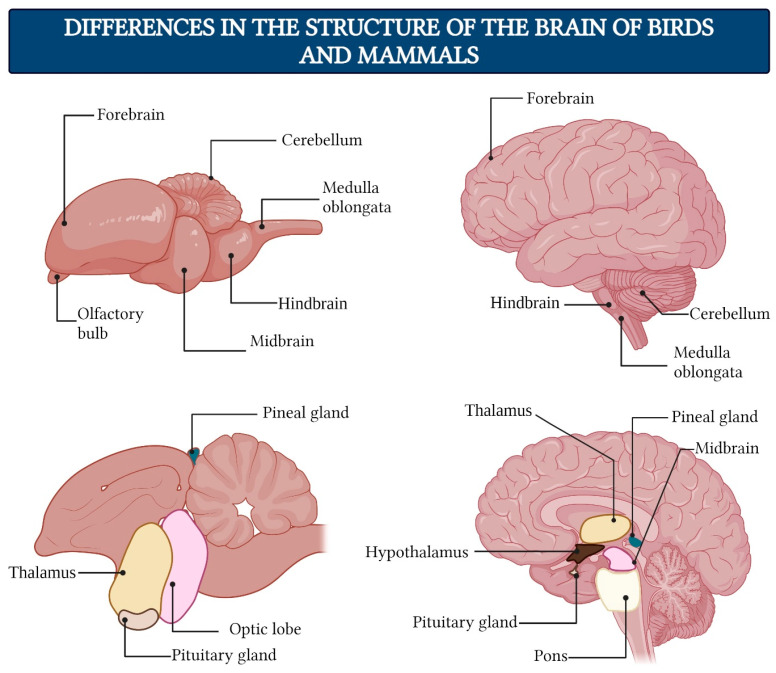
Characteristic morphological features of the brain of birds and mammals (using the example of humans) In mammals, the pallium gives rise to a layered neocortex, whereas in birds it forms clustered gray-matter nuclei (e.g., hyperpallium, nidopallium). Importantly, despite these differences in macroscopic architecture, both taxa utilize convergently evolved neuronal mechanisms—high associative neuron counts, prefrontal-like pallial regions, dense dopaminergic inputs and dynamic working memory circuits—to achieve similarly sophisticated cognitive and conscious functions.

Throughout the progressive evolution of mammals, many higher brain functions shifted from subcortical to cortical regions. Many cognitive neuroscientists believe that both the cerebrum and the neocortex are critical for complex cognition (Figure 7). However, recent comparative research shows that corvids and parrots, which possess noncortical brains weighing only 1–25 g, demonstrate cognitive abilities on par with great apes such as chimpanzees, whose brains weigh around 400 g [294]. Four characteristics have been identified as possibly necessary for complex cognition in birds: (1) a large number of associative pallial neurons; (2) a region analogous to the prefrontal cortex in mammals; (3) dense dopaminergic innervation of associative brain regions; and (4) dynamic neurophysiological foundations for working memory. These four neuronal characteristics evolved convergently, making them likely “hard-to-replace” mechanisms for supporting complex cognition in birds [295]. The last common ancestor of modern birds and mammals lived around 324 million years ago, and over time, these two taxa developed completely different forebrain structures [295].

In mammals, the dorsal pallium evolved into the cerebral cortex, most of which is isocortical. In contrast, the homologous avian dorsal pallium became the hyperpallium, which has a nuclear form. Most of the remaining avian pallial nuclei are located beneath the lateral ventricle and are collectively called the dorsal ventricular ridge (DVR) [270]. This area lacks a direct analog in mammals, and its hypothesized homologies with various mammalian pallial components remain a subject of debate. It appears that the distribution of gray-matter cells responsible for cognitive functions in the form of the cortex in mammals offers significant advantages only when there is a substantial number of these cells. This facilitates communication between these cells and other brain compartments through efficiently arranged neural fibers in the form of white matter in the cerebral cortex.

Therefore, the emergence of human consciousness is the result of a long evolutionary process in the development of higher nervous activity in ancestral species. Over time, humans created a global cultural environment, crossing the threshold between biological consciousness and a qualitatively different state—individual and societal consciousness linked to the culture of the civilized world.

Overall, the evolution of consciousness and cognitive functions is characterized not only by a relatively continuous and sequential development of the nervous system but also by discrete systemic transitions. The first such transition occurred approximately 3.6 billion years ago with the emergence of life on Earth, based on the informational matrix of individual genotypes and the population gene pools of the biosphere. This development laid the foundation for the eventual emergence of biological consciousness. At present, we are witnessing the second “Great” qualitative transition, associated with the transformation of the biosphere into the anthropogenic noosphere—the sphere of human reason—characterized by supra-biological laws based on the informational matrix of collective human consciousness [296,297,298,299,300,301,302,303,304,305]. At the same time, conditions are accumulating for what may be an even more fundamental transition—the potential emergence of an autonomous system of artificial (technogenic) intelligence, particularly one associated with computer networks [305,306,307,308,309].

### 4.3. Evolution of Human Consciousness

The question of when and in what form consciousness emerges during ontogeny remains far from resolved. However, recent research indicates that certain forms of consciousness may be present in the fetus by the third trimester of pregnancy [310]. The formation of socialized (culture-dependent) consciousness, on the other hand, begins around the first year of a child’s life [310].

In developmental biology, it is recognized that while ontogeny (the development of an individual organism) does not directly recapitulate phylogeny (the evolutionary history of its species), there are significant connections between the two. The historical concept that “ontogeny recapitulates phylogeny”, proposed by Haeckel, suggested that an organism’s developmental stages mirror the sequential stages of its evolutionary ancestors. However, this notion has been largely discredited in modern biology [311,312,313].

Contemporary evolutionary developmental biology acknowledges that developmental processes can reflect evolutionary relationships without implying a direct recapitulation. For example, certain embryonic features may be conserved due to shared genetic pathways and developmental constraints, providing insights into common ancestry. This perspective, sometimes referred to as “deep homology”, highlights how similar genes and developmental mechanisms can produce analogous structures in different species [314]. However, the connection between evolution at the genotypic and phenotypic levels is surprisingly fluid, multi-layered, and complex. As a result, homology between processes cannot always be traced in terms of homology between genes [315]. Therefore, developmental changes used to explain evolutionary transformations must be placed in their actual historical sequence. Such an analysis leads to the conclusion that the neurobiological structure of the vertebrate CNS is evolutionarily ancient and highly conserved across species, and that the fundamental neurophysiological mechanisms supporting consciousness in humans can be found in the earliest stages of vertebrate brain evolution [316,317]. At the same time, it is necessary to consider both general interspecies features, such as the presence of parallel mechanisms for sequential vocal learning based on auditory (input) and motor (output) pathways, and species-specific characteristics of this process in humans [318].

This principle likely applies to the early stages of human consciousness development as well. The development of consciousness in ontogeny is associated with an increase in the number of synaptic connections, particularly in the cerebral cortex. The prefrontal cortex of the frontal lobes, the site of higher cognitive functions, continues to develop into adulthood, approximately until the third decade of life [319].

Phylogenetically, humans differ from their evolutionary predecessors not only in having a more developed brain but also in possessing advanced upper limbs capable of making tools. In addition, humans exhibit a high capacity for symbolic and abstract thinking, as well as the ability to use language to express subjective experiences in communication, with inner speech serving as one of the mechanisms of thought. It is worth noting that many of these properties of consciousness are present, to some extent, in highly mentally organized animals, including the ability of bottlenose dolphins, for example, to use learned vocal signals to coordinate their behavior and cooperate with conspecifics [242]. In humans, however, the rhythm and synchronization of speech possess a hierarchical temporal structure (phonemes, syllables, words, sentences). Interestingly, animals from various taxonomic groups also exhibit a hierarchical temporal structure in vocalizations. These animals can produce and perceive vocalization patterns with temporal and rhythmic features that correspond to the human sense of rhythm [320].

Moreover, it is well established that certain species of monkeys and birds can use primitive tools, such as for extracting food [321]. Additionally, the ability to learn abstract concepts is observed in many species of mammals and birds [268,276,322]. Therefore, human consciousness differs from that of highly mentally organized animals not through entirely unique traits, but rather through qualitative and systemic distinctions, which led to the transition from a biological to a social system. Such a transition could not have been a singular event, but rather required a relatively long period for the accumulation of rudimentary cultural potential and changes in the collective consciousness of prehistoric human communities.

Thus, the progressive evolution of species organization and complex animal behavior programs, based on the increasing role of acquired memory, predetermined the emergence of a governing functional system—subjective consciousness. This governing system became more complex than the programs it controlled, which, in turn, required significant growth and complexity in the functions, structure, and resources of the brain. It appears that the emergence of consciousness in animals and humans is not a discrete process.

**Figure 8:**
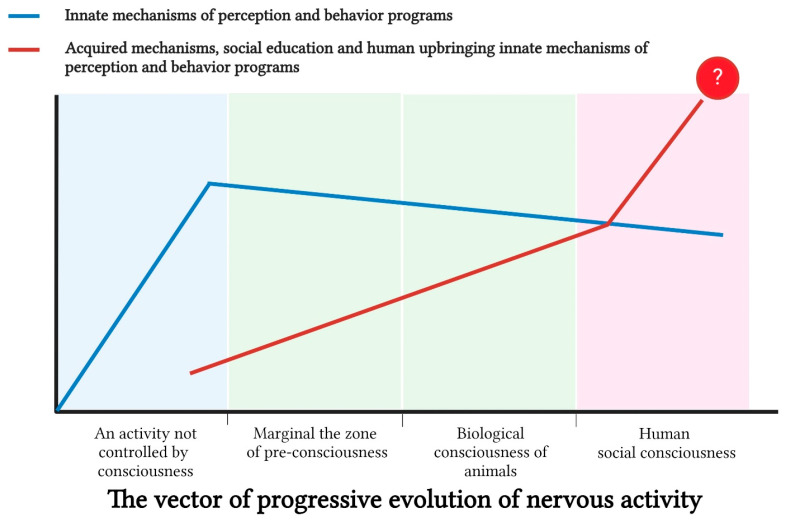
Conditional stages in the evolution of subjective consciousness. Note: The emergence of consciousness appears to be a natural consequence of the evolution of autonomous, self-replicating, and informationally self-developing (progressively advancing) systems. Within this framework, the evolution of consciousness can be characterized by two relatively discrete systemic qualitative transitions: (1) The Origin of Life on Earth (~3.6 billion years ago)—This marked the emergence of the fundamental laws governing the biosphere, which complemented but did not override the physical laws of material existence. The emergence of living matter inevitably led to the evolutionary development of sentient beings endowed with consciousness. This process was relatively gradual and continuous, involving: the formation of individual nerve cells; the development of a nervous system based on innate memory mechanisms; the emergence and progressive expansion of acquired memory and increasingly complex behavioral programs; the transitionary stage of proto-consciousness; and, ultimately, the development and refinement of biological consciousness in animals, culminating in the appearance of early humans. (2) The Formation of Human Cultural Environments—The second qualitative transition occurred with the development of human societies governed by distinct cultural laws. In this context, the progress of informational systems became decoupled from changes in the genetic matrix of humanity’s gene pool and instead relied on modifications in the informational matrix of collective consciousness. This matrix now includes an external informational framework comprising technogenic databases and artificial intelligence carriers. The presence of such an external informational framework in anthropogenic social systems raises the question of whether another “Great Leap” in the evolution of consciousness is imminent—one potentially associated with the emergence of an independent subjectivity within informational technogenic systems, as they progress toward self-sufficiency and autonomy from human influence.

From this perspective, the evolution of consciousness can be tentatively divided into several stages: (1) the gradual accumulation of working and long-term acquired memory, as well as the systematization of innate and acquired mechanisms of perceptual and behavioral functional systems; (2) the formation of a transitional zone in the form of preconsciousness, with certain features of this state observable not only in vertebrates but also in highly organized invertebrates such as cephalopods; (3) the emergence of clear signs of subjectivity in highly organized warm-blooded animals (birds and mammals); (4) the appearance of human socialized, culturally mediated consciousness, which has extended its external framework (through technological memory and artificial intelligence) beyond the gene pool and phenotype of the biosphere (see Figure 8). As the foundational system-forming factor of society, the collective consciousness of humanity, on the whole, is harmonized with the exponential growth and continuous complexity of society’s cultural environment.

What predictions can be made based on this proposed logic of the evolution of consciousness in living systems?

## 5. What’s Next?

It seems that by now, the development of the Earth’s biosphere has reached its limit. However, humanity has managed to overcome these limitations by creating a cultural environment within the altered biosphere (noosphere). For a long time, the acquired memory of the nervous and immune systems, and later biological consciousness, acted as extensions of the genetic matrix of the biosphere [169,323,324,325]. Meanwhile, the driving force behind societal development has not been the dynamic changes in the human gene pool, but rather the interaction between public consciousness and the cultural environment, where the former functions as an informational matrix, analogous in function to the genetic matrix of the biosphere.

**Figure 9:**
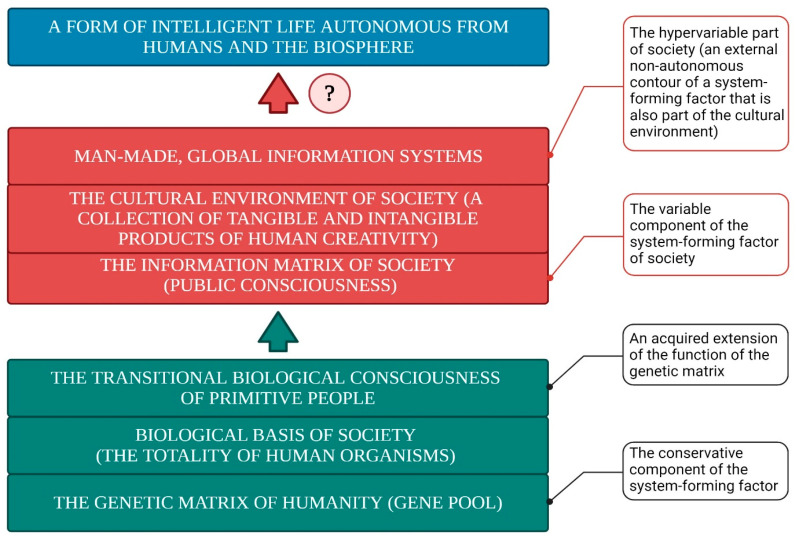
Major stages of the evolution of intelligence in living systems. Note: Figure 9 illustrates the qualitative transition from biological consciousness to the social consciousness of human civilizations, including the relatively prolonged transitional period of the Paleolithic era among early humans. This transition was marked by the formation of the noosphere, whose system-forming factor is based not only on the genetic matrix of humanity’s gene pool but, to an even greater extent, on the informational matrix of collective human consciousness. For a long time, biological consciousness in animals merely supplemented the genetic matrix of the biosphere, serving as a phenotypic extension of genetic information. However, the social consciousness of human societies established the noosphere as a system-forming factor—an entity that currently includes the technosphere, which, at present, lacks independent subjectivity. Nevertheless, the evolutionary logic of self-organizing informational systems suggests the potential for yet another “Great Leap”—the emergence of an autonomous technogenic form of life and intelligence, independent of human influence.

At the same time, human collective consciousness, like the genetic matrix of the biosphere, has an external, non-autonomous circuit that significantly expands its functions, but in the form of rapidly changing technogenic information systems. Thus, the genetic matrix, as the biological foundation of humanity, remains the most conservative part of the system-forming factor of society. Furthermore, the presence of innate programs of consciousness, the natural basic capacities of the human brain, the limited lifespan of individuals, communication challenges, and other biological limitations act as restraining mechanisms for the progressive development of collective consciousness and, consequently, the technogenic component of society (Figure 9).

We believe that the task of science, even now, is to study the specific mechanisms underlying the aforementioned qualitative transformation of information systems, with the aim of ensuring more favorable outcomes for humanity during this transition. Insights from ancestral system-forming factor of consciousness architectures—such as subcortical arbitration circuits—illuminate why default-mode network (DMN) disruptions in Alzheimer’s and salience-network imbalances in schizophrenia undermine the integration of memory, emotion, and executive control [326,327]. By tracing these network motifs back to early vertebrate evolution, we propose that disease phenotypes reflect partial regressions or dysregulations of the ancestral variants of biological consciousness. Even now, several vectors of societal development can be identified that, at a certain stage of quantitative change, may lead to systemic qualitative transformations, including the following:

- Increasing urbanization, globalization, and unification of all key areas of human life, leading to a reduction in the diversity of individual subsystems within society, each with its own characteristics of collective consciousness.
- The creation of unmanned production and logistics systems based on robotics and the implementation of AI; the displacement of humans from socially useful spheres of activity [328].
- The automation and AI control of all spheres of human life (medicine, education, science, art, entertainment, household and community services, legal support, family communication, etc.) [328,329,330,331,332,333].
- The rapid development of global data centers for accumulating and analyzing information through AI, such as for the use of language models like ChatGPT, including in medicine [334].
- The self-development of computer programs based on artificial neural network technologies and their hybridization with other methods [335].
- The use of AI as a tool that can complement, and potentially replace, human creativity [336,337,338,339].
- The hybridization of AI approaches reflects both centralized (mammalian-like) and decentralized (cephalopod-like) neural processing, which could lead to hybrid intelligence models integrating the strengths of both approaches.
- The presence of computational thinking—the ability to reformulate and solve problems in ways that can be executed by computers, declared a foundational capability for the development of intelligent systems in the 21st century [340].
- The spread of trans-humanist ideology, aimed at altering and enhancing the natural characteristics of humans through biological, technological, and cognitive modifications, with the ultimate goal of transforming humans into a “new human” [341,342,343]. Additionally, post-humanist ideology, whose conceptual foundations include scientific discoveries that have blurred the boundaries between humans and other living beings; the development of artificial intelligence; changes in perceptions of humanity; and the hybridization of humans with other living organisms and machines [344,345]. While trans-humanism seeks to overcome human intellectual and physical limitations, post-humanism seeks to surpass humanism itself by creating the “post-human” [346,347].

Scientific research should focus on the changes along the axis of the gene pool of society—collective consciousness—AI. It is now well established that human cognitive abilities depend on their genetics, with a complex interplay between many genes and the environment that affects their function. The genetic principles of this relationship include pleiotropy (each gene influences many traits), polygenicity (many genes influence each trait), additivity (the cumulative action of genes without dominance of specific genes), epistasis (the interaction of non-allelic genes), and many other genetic mechanisms, making genetic control over consciousness and thinking highly complex and internally contradictory [228,229,230,348,349,350]. The genetic diversity of these genes, within the framework of population natural polymorphism, in turn, accounts for the diversity of individual cognitive abilities, which defines the adaptive capacity of collective consciousness.

In the 20th century, several countries attempted to improve the population gene pool under the banner of “eugenics” and “racial biology”, which included forced sterilization, involuntary euthanasia, and even utopian projects to create human–ape hybrids. This led to the discrediting of eugenics as a scientific field within genetics [351,352,353]. Currently, non-directive counseling methods, preserving the autonomy of the counselee, have helped to form approaches and outline the goals and limitations of the new profession of “genetic counseling” [354]. Additionally, future improvements, such as reprogramming procedures and gene editing technologies, are now being discussed [354,355]. These considerations extend to ethical dilemmas related to human cloning, prompting extensive reflection on values such as human dignity, autonomy, and safety.

Equally important is the scientific direction of assessing the potential negative impacts of AI on human consciousness. For instance, there is already data showing the negative influence of AI on decision-making abilities, laziness, and privacy concerns among university students [356]. AI models can reinforce and amplify human biases if they are trained on biased data, leading to a lack of critical thinking among people [357]. Additionally, the improper use of narrow AI poses a threat to human health by increasing the possibilities for control and manipulation of people [358]. It is no coincidence that recent attention has been focused on legal and human rights issues related to AI [359]. As AI models, especially those inspired by decentralized architectures, grow more complex, it is crucial to assess how these systems will influence human cognition and consciousness. It is also important to develop tactics and strategies for reverse integration, namely, the integration of humans into AI systems. This mirrors the inherent risks of centralized control in AI, where excessive attempts to impose strict oversight might limit the adaptive potential of AI systems. On the other hand, a high degree of decentralization of this influence could lead to humans losing control over integrated AI systems.

### 5.1. Positioning Our Subjective System-Forming Model Among Evolutionary Naturalisms

Our model and Feinberg & Mallatt’s neurobiological naturalism share a common evolutionary foundation: both view consciousness not as an a priori attribute of life but as a weakly emergent property of increasingly complex nervous systems shaped by natural selection [360,361,362,363,364]. At the same time, in our view, the Cambrian evolutionary explosion (542–485 million years ago) predetermined the emergence of distinct functions of consciousness in arthropods, a transitional stage—preconsciousness—in cephalopod mollusks and many vertebrate species, the rise of a biological level of consciousness in certain highly organized birds and mammals, and, ultimately, the appearance of culturally mediated, supra-biological consciousness in human societies. Whereas Feinberg & Mallatt delineate successive phylogenetic tiers—from life’s basic embodied properties to reflexive neural circuits and, finally, to specialized neural features that close the explanatory gaps of referral, unity, causation and qualia—our theory introduces subjectivity itself as an active, system-forming factor (SFF) [13,14,15,16,17]. In this framework, the emergent “subjective I” orchestrates information flow from sensory filtering through causal modeling to future prediction, directing both working and long-term memory to resolve competing instinctual programs. This arbitration mechanism, absent from purely neurocentric accounts, can be formalized quantitatively in terms of information throughput and priority weighting, thereby extending the anatomical correlates of SNFs into explicit computational models.

Moreover, our theory provides a unique evolutionary perspective on the distribution of consciousness. We propose that, at present, it is questionable to speak of subjectivity in arthropods and primitive ancestral vertebrates. Rather, it is more appropriate to discuss certain general functions of their central nervous systems that are shared with the conscious activities of advanced vertebrates exhibiting signs of a “self”. For example, the geolocation ability in bees, which is based on acquired memory, demonstrates such a function—but this does not imply awareness of these functions.

Consciousness, in our view, evolved independently in vertebrates, many arthropods, and cephalopods—a pattern that exemplifies constrained multiple realizability. In this framework, similar mental functions emerge from distinct neural architectures, yet remain bounded by shared physical, physiological, and historical constraints [17]. In stark contrast to highly speculative frameworks such as the Cellular Basis of Consciousness, we maintain that true phenomenal experience requires a nervous system and a brain of threshold complexity, rather than being a fundamental property of individual cells [360].

Crucially, our approach traces an additional human transition: the cultural emergence of consciousness via symbolic systems and collective meaning-making, which externalize and accumulate experience beyond the biological matrix. In this noospheric phase, subjective architectures operate not only within individual brains but across technological media and shared symbolic repositories, laying the groundwork for a possible future emergence of nonbiological subjectivity in autonomous artificial systems. By shifting the focus from neural substrates alone to the functional organization of consciousness under SFF guidance, our model both complements and extends Feinberg & Mallatt’s theory, forging an integrative path that links evolutionary biology, quantitative information dynamics and socio-cultural exaptation.

### 5.2. Cultural Exaptation and Technogenic Frontiers

Human consciousness transcends biological constraints through cultural scaffolding, in which symbolic systems—language, writing, and digital media—form a noospheric feedback loop. This concept, initially articulated by Vernadsky (1945) and Teilhard de Chardin (1959), facilitates supra-biological memory through the externalization of knowledge into texts, databases, and artificial intelligence systems [298,301,302,303,304,305,361,362]. It supports collective meaning-making via shared narratives that shape individual subjectivity and modulate prefrontal–parietal neural connectivity [363,364,365,366]. Additionally, cultural practices drive neuroplastic reshaping: for example, literacy acquisition reorganizes phonological and orthographic processing within occipito-temporal regions [367,368].

Empirical studies confirm this cultural plasticity. Early tool-use training reshapes frontoparietal pathways, evidencing deep-time bio-cultural feedback [369]. Literacy-induced cortical recycling is demonstrated by increased left ventral occipito-temporal activation in previously illiterate adults after literacy training [368]. Musical expertise enlarges the auditory cortex and strengthens fronto-temporal connectivity in professional musicians [370]. Early bilingualism increases gray-matter density in the anterior cingulate and dorsolateral prefrontal cortex, correlating with enhanced conflict-resolution abilities [371].

Brain–computer interfaces (BCIs) represent the technogenic frontier, extending subjectivity into synthetic substrates. Early non-invasive BCIs translated EEG rhythms into external commands [372], while invasive systems allowed precise intracortical decoding [373,374]. Recent architectures combining EEG, NIRS, and deep learning—such as LSTM, convolutional, and temporal convolutional networks—enable high-fidelity motor prediction and adaptive closed-loop control [374,375]. These technologies are increasingly being applied in clinical neurology, offering therapeutic gains in stroke, spinal cord injury, and disorders of consciousness [376].

### 5.3. Emergence of Non-Human-Generated Cultural Substrates

A qualitatively new phenomenon is now occurring: the emergence of non-human-generated cultural substrates. For millennia, all learning, analysis, and cultural development were grounded in artifacts, narratives, and data produced by human minds—be it epic poetry, music, philosophy, or scientific treatises. Even computational tools historically served only as interfaces for manipulating human-created content.

Now, for the first time, the raw materials of cognition—texts, images, music, even scientific hypotheses—are being produced autonomously by machine learning models and generative algorithms. These outputs are not merely recombinations of existing human knowledge, but often constitute genuinely novel structures, patterns, or ideas that emerge from the internal logic of artificial architectures [331]. This shift inaugurates a new axis of co-evolution: not only do humans learn from machine-generated content, but artificial systems themselves adapt to human responses, forming recursive feedback loops in which meaning and subjectivity are distributed across human and non-human agents.

This development has profound implications for the formation of subjectivity and the future of hybrid consciousness. Historically, the subjective self was shaped through the assimilation of human cultural codes; now, hybrid forms of cognition arise in which meaning structures are co-constructed by both biological and artificial systems, some of which lack intentionality or sentience in the traditional sense. These challenges established models of epistemology, education, and trust: humans must now interpret, validate, and integrate meanings generated by systems that do not share human experience or intentionality.

A striking recent example is provided by Kobak et al. (2025), who demonstrated that large language models (LLMs) have already left a measurable stylistic fingerprint on scientific writing: at least 13.5% of biomedical abstracts in 2024 were processed with LLMs, with this proportion reaching up to 40% in some subfields [377]. This unprecedented impact of machine-generated text on the core substrate of scientific communication illustrates how non-human agents are now actively shaping the evolution of knowledge and meaning in real time [377].

The emergence of non-human-generated cultural substrates thus marks a radical transformation in the sources and dynamics of subject formation, potentially leading to new forms of hybrid or distributed consciousness. This transition demands new frameworks for understanding agency, authorship, and the evolution of meaning in the age of artificial intelligence.

## 6. Conclusions

This review documents the evolutionary plausibility—not inevitability—of consciousness. While phylogenetic data reveal conserved neural architectures enabling consciousness, they cannot prove its inevitability. The “hard problem” of why specific neural dynamics generate subjective experience remains unresolved. Convergent neural architectures (e.g., thalamo-cortical loops in mammals, vertical lobes in cephalopods) enable similar cognitive functions under equivalent selective pressures. However, three unresolved boundaries persist:

- The Complexity Threshold: No current model predicts why octopuses (500 M neurons) exhibit goal-directed cognition while similar-complexity AI systems do not.
- The Subjectivity Gap: SFF mechanisms (e.g., conflict arbitration) are empirically tractable in vertebrates but lack computational formalization for artificial systems.
- The Technogenic Leap: Cultural scaffolding extends consciousness beyond biology, but whether AI can autonomously replicate SFF dynamics remains untested. Future research must quantify SFF dynamics across phylogenetic scales while developing cross-species assays for subjective arbitration.

Future research must quantify SFF dynamics across phylogenetic scales while developing cross-species assays for subjective arbitration.

In this framework, subjectivity functions as a system-forming factor (SFF) implemented through hierarchical neural architectures—integrating sensory filtering, memory prioritization, and behavioral arbitration without centralized “controllers”. Subjectivity is therefore not merely an attribute of consciousness but its core organizational principle. Although the precise phylogenetic emergence of subjectivity remains uncertain, convergent evidence indicates that mentally advanced mammals and certain bird species exhibit clear signs of self-awareness [288,310,378].

Biological consciousness emerged gradually as multilevel architectures—beginning with unicellular information systems, progressing through proto-conscious invertebrates, and culminating in vertebrate systems—provided a selective edge for resolving conflicting drives. In highly organized taxa (e.g., birds, mammals), subjectivity integrates sensory, mnemonic, and motivational modules into a unitary “self”. In Homo sapiens, this system extended beyond biology into the culturally mediated noosphere, and the stage is set for a potential technogenic transition to artificial subjectivity.

We also acknowledge the quantum-delocalized information framework of Poznanski et al. (2025) as an intriguing molecular-quantum account of consciousness emergence in neuropil micro-cavities; however, given its current tentative experimental support and its orthogonality to our systems-level subjectivity framework, we mention it here only for completeness [25,378,379]. This paper remains centered on macro-scale, evolvable architectures and subjective arbitration mechanisms that can be tested across taxa; detailed protein-level quantum coherence falls outside our current scope [25,378,379].

In Section 5.1, we explicitly compared our framework to Feinberg & Mallatt’s neurobiological naturalism, showing that while both theories share core tenets—weak emergence, rejection of panpsychism and anthropocentrism, and constrained multiple realizability—our model advances the field by elevating subjectivity to the role of an active system-forming factor, formalizing arbitration and memory dynamics, and extending the evolutionary narrative into the cultural noosphere and potential technogenic consciousness. These refinements clarify how our model both complements and extends existing evolutionary theories, positioning it as an integrative pathway from neural origins to cultural exaptation and beyond.

## Data Availability

No new experimental data were created.
